# The impact of selenium administration on severe sepsis or septic shock: a meta-analysis of randomized controlled trials

**DOI:** 10.4314/ahs.v21i1.36

**Published:** 2021-03

**Authors:** Lin Kong, Qing Wu, Bo Liu

**Affiliations:** 1 Department of Clinical Nutrition, Children's Hospital of Chongqing Medical University, Chongqing 400014, China; 2 Department of Pharmacy, Children's Hospital of Chongqing Medical University, Chongqing 400014, China; 3 Department of Gastroenterology, Children's Hospital of Chongqing Medical University, Chongqing 400014, China; 4 Ministry of Education Key Laboratory of Child Development and Disorders, National Clinical Research Center for Child Health and Disorders, China International Science and Technology Cooperation base of Child development and Critical Disorders, Chongqing Key Laboratory of Pediatrics, Children's Hospital of Chongqing Medical University Chongqing 400014, China

**Keywords:** Selenium administration, septic shock, randomized controlled trials

## Abstract

**Introduction:**

The efficacy of selenium administration to treat severe sepsis or septic shock remains controversial. We conduct a systematic review and meta-analysis to explore the impact of selenium administration on severe sepsis or septic shock.

**Methods:**

We search PubMed, EMbase, Web of science, EBSCO, and Cochrane library databases through May 2020 for randomized controlled trials (RCTs) assessing the effect of selenium administration on severe sepsis or septic shock. Meta-analysis is performed using the random-effect model.

**Results:**

Five RCTs involving 1482 patients are included in the meta-analysis. Overall, compared with control group in septic patients, selenium administration is not associated with reduced 28-day mortality (RR=0.93; 95% CI=0.73 to 1.19; P=0.58), but results in substantially decreased all-cause mortality (RR=0.78; 95% CI=0.63 to 0.98; P=0.03) and length of hospital stay (MD=-3.09; 95% CI=-5.68 to -0.50; P=0.02).

**Conclusion:**

Selenium administration results in notable decrease in all-cause mortality and length of hospital stay, but shows no substantial influence on the 28-day mortality, length of ICU stay, duration of vasopressor therapy, the incidence of acute renal failure, adverse events, and serious adverse events for septic patients.

## Introduction

Sepsis and septic shock remain a major health care problem, and the incidence of severe sepsis has increased by 1.5 % ever year^1^–^3^. Severe sepsis has emerged as the leading cause of hospitalization and accounts for 2 % of all hospital admission, 59 % of the patients in intensive care unit (ICU)^4^, ^5^. The mortality of severe sepsis and septic shock still remains unacceptably high despite of the treatment development^6^–^8^.

Inflammatory cytokines and oxidative stress released during sepsis are high in septic patients, and their concentrations have some association with severity and evolution of organ dysfunctions^9^–^11^. Patientswith severe sepsis have oxidative stress that may cause multi-organ failure and death^12^. Decreased plasma selenium levels are found to be associated with excess mortality^13^. Intravenous administration of selenium is reported to restore the activity of glutathione peroxidase, attenuates oxidative stress, and may improve survival in septic patients^14^–^16^. In contrast, a cocktail of antioxidants and vitamins containing 800 ug of selenium do not decrease 28-day mortality in patients receiving mechanical ventilation^17^. This increase in the concentration of selenium is associated with the reduction in the oxidative stress and sequential organ failure assessment (SOFA) score^15^.

The use of selenium has not been well established in septic patients. Recently, several studies on the topic have been published, and the results have been conflicting^18^–^20^. With accumulating evidence, we therefore perform a systematic review and meta-analysis of RCTs to investigate the efficacy of selenium administration to treat septic patients.

## Materials and methods

Ethical approval and patient consent are not required because this is a systematic review and meta-analysis of previously published studies. The systematic review and meta-analysis are conducted and reported in adherence to PRISMA (Preferred Reporting Items for Systematic Reviews and Meta-Analyses)^21^.

### Search strategy and study selection

Two investigators have independently searched the following databases (inception to May 2020): PubMed, EMbase, Web of science, EBSCO, and Cochrane library databases. The electronic search strategy is conducted using the following keywords: s/span>elenite or selenium, and sepsis or septic. We also check the reference lists of the screened full-text studies to identify other potentially eligible trials.

The inclusive selection criteria are as follows: (i) population: patients diagnosed with severe sepsis, septic shock; (ii) intervention: selenium administration; (iii) comparison: placebo; (iv) study design: RCT.

### Data extraction and outcome measures

We have extracted the following information: author, number of patients, age, body mass index, Acute Physiologic and Chronic Health Evaluation II (APACHE II) scores etc. The data have been extracted independently by two investigators, and discrepancies are resolved by consensus. We also contact the corresponding author to obtain the data when necessary. No simplifications and assumptions are made. The primary outcomes are 28-day mortality and all-cause mortality. Secondary outcomes include length of ICU stay, length of hospital stay, duration of vasopressor therapy, the incidence of acute renal failure, dverse events, and serious adverse events.

### Assessment for risk of bias

We used the risk of bias tool to assess the quality of individual studies in accordance with the Cochrane Handbook for Systematic Reviews of Interventions^22^, and the sources of bias included selection bias, performance bias, attrition bias, detection bias, reporting bias, and other potential sources of bias. The bias was evaluated and rated: high, low, and unclear^23^. Two investigators independently searched articles, extracted data, and assessed the quality of included studies. Any discrepancy was solved by consensus.

### Statistical analysis

We estimate the mean difference (MD) with 95% confidence interval (CI) for continuous outcomes (length of ICU stay, length of hospital stay, and duration of vasopressor therapy) and the RR with 95% CIs for dichotomous outcomes (8-days mortality and all-cause mortality, the incidence of acute renal failure, adverse events, and serious adverse events). A random-effects model is used regardless of heterogeneity. Heterogeneity is reported using the I2 statistic, and I2 > 50% indicate significant heterogeneity^24^. Whenever significant heterogeneity is present, we search for potential sources of heterogeneity via omitting one study in turn for the meta-analysis or performing subgroup analysis. Publication bias is not evaluated because of the limited number (<10) of included studies. All statistical analyses are performed using Review Manager Version 5.3 (The Cochrane Collaboration, Software Update, Oxford, UK).

## Results

### Literature search, study characteristics and quality assessment

**Figure. 1:**
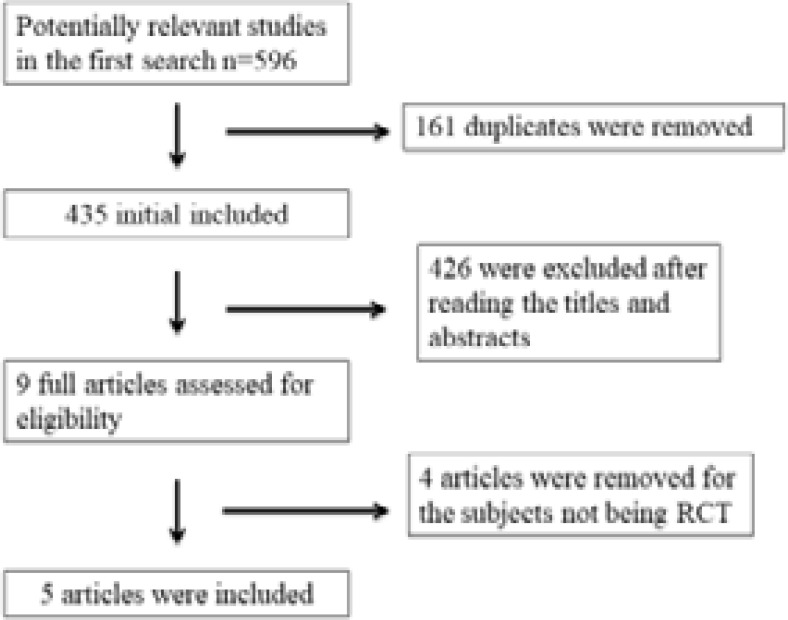
Flow diagram of study searching and selection process.

A detailed flowchart of the search and selection results is shown in Figure 1. 596 publications are searched after the initial search of databases. 161 duplicates and 426 papers are excluded after checking the titles/abstracts. 4 studies are removed because of the study design25–28 and five RCTs that meet our inclusion criteria are finally included in the meta-analysis^16^, ^18^–^20^, ^29^.

The baseline characteristics of the five eligible RCTs in the meta-analysis are summarized in Table 1. The five studies are published between 2004 and 2017, and sample sizes range from 41 to 1089 with a total of 1482. The methods using selenium in each RCT are different, detail in Table 1.

**Table 1:** Characteristics of included studies

NO.	Author	Selenium group						Control group					
		Number	Age (years)	Male (n)	Body mass index (kg/m^2^)	APACHE II score	Methods	Number	Age (years)	Male (n)	Body mass index (kg/m^2^)	APACHE II score	Methods
1	Chelkeba 2017	29	35(17–82), median (IQR)	22	-	17±4.3	2 mg intravenous bolus followed by 1.5 mg intravenous infusion daily for 14 days	25	41(19–82), median (IQR)	22	-	16.4±4.0	Matched placebo
2	Bloos 2016	543	-	345	-	-	an initial intravenous loading dose of sodium selenite, 1000 ug, followed by a continuous intravenous infusion of sodium selenite, 1000 ug, daily until discharge from the intensive care unit, but not longer than 21 days	546	-	346	-	-	Matched placebo
3	Forceville 2007	31	66±14	20	-	-	sodium selenite for 10 days (4,000 ug on the first day, 1,000 ug/day on the nine following days)	29	69 ± 12	18	-	-	Matched placebo
4	Angstwurm 2007	116	63.9±13.8	86	27.1±6.8	-	1000 ug of sodium-selenite as a 30-min bolus injection, followed by 14 daily continuous infusions of 1000 ug intravenously	122	65.3±14.1	76	26.7±5.0	-	Matched placebo
5	Angstwurm 2004	20	54.3±4.9	16	21±1.5	17±1.3	sodium selenite (500 ug/day for the first 3 days, reducing to 250 and then 125 ug/day every 3 days)	21	58.5±5.2	13	24±1.4	19±1.1	Matched placebo

APACHE II: Acute Physiologic and Chronic Health Evaluation II.

Among the five studies included here, four studies report the 28-day mortality^16^, ^18^–^20^, all-cause mortality^16^, ^19^,^20^, length of ICU stay^16^, ^18^, ^20^, length of hospital stay18, 20, duration of vasopressor therapy18–20, the incidence of acute renal failure^16^, ^19^, adverse events^16^, ^18^, and serious adverse events^18^, ^20^.

### Assessment of risk of bias

**Figure. 2:**
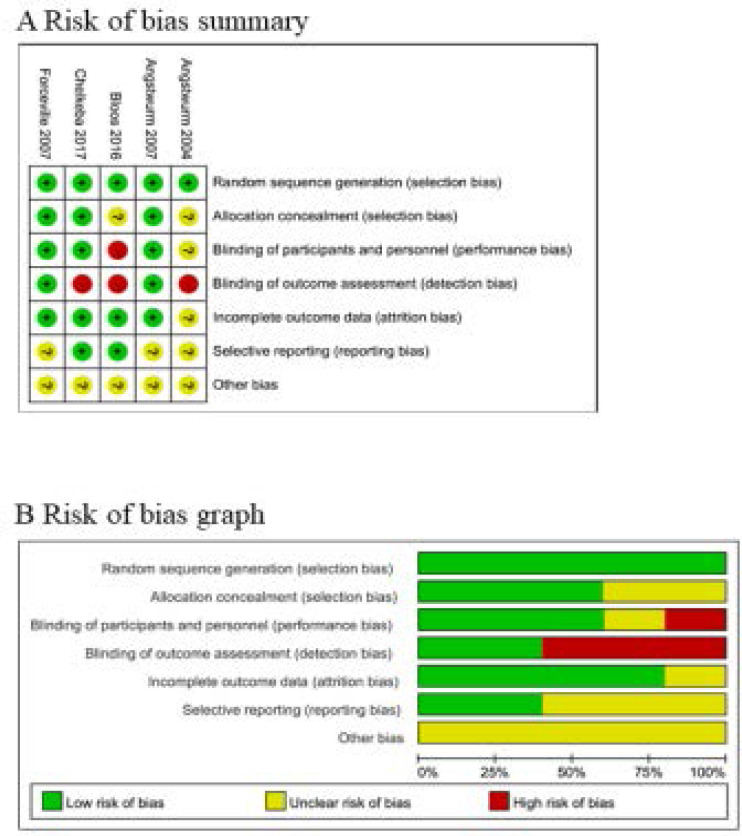
Risk of bias assessment. (A) Authors' judgments about each risk of bias item for each included study. (B) Authors' judgments about each risk of bias item presented as percentages across all included studies.

Risk of bias analysis (Figure 2) showed that one study had high risk of bias because it was a single-blind RCT^19^,^29^, and another trial had high risk of bias due to the non-blindness^18^.

### Primary outcome: 28-day mortality and all-cause mortality

**Figure. 3:**
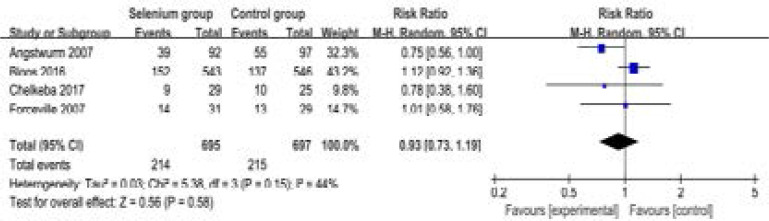
Forest plot for the meta-analysis of 28-day mortality.

These two outcome data are analyzed with the random-effects model, and the pooled estimate of the four included RCTs suggested that compared to control group in septic patients, selenium administration shows no substantial influence on the 28-day mortality (RR=0.93; 95% CI=0.73 to 1.19; P=0.58), with low heterogeneity among the studies (I2=44%, heterogeneity P=0.15) (Figure 3).

**Figure. 4:**
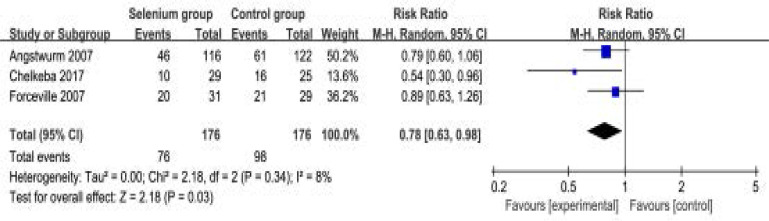
Forest plot for the meta-analysis of all-cause mortality.

In contrast, selenium administration is found to significantly reduce all-cause mortality (RR=0.78; 95% CI=0.63 to 0.98; P=0.03) after pooling the results of three included RCTs, with low heterogeneity among the studies (I2=8%, heterogeneity P=0.34) (Figure 4).

### Sensitivity analysis

Low heterogeneity is observed among the included studies for the 28-day mortality and all-cause mortality. Thus, we do not perform sensitivity analysis by omitting one study in each turn to detect the source of heterogeneity.

### Secondary outcomes

**Figure. 5:**
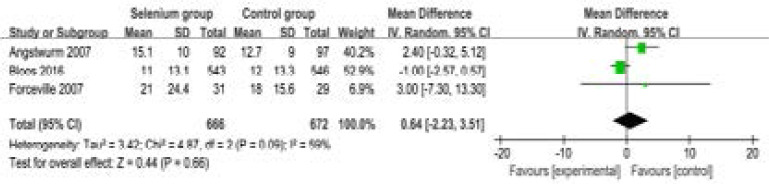
Forest plot for the meta-analysis of length of ICU stay (day).

**Figure. 6:**
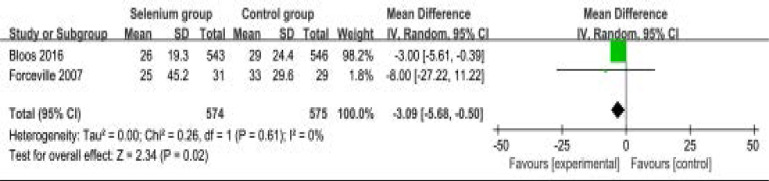
Forest plot for the meta-analysis of length of hospital stay (day).

Compared to control group in septic patients, selenium administration has no remarkable impact on length of ICU stay (MD=0.64; 95% CI=-2.23 to 3.51; P=0.66; Figure 5), but leads to shorter length of hospital stay (MD=-3.09; 95% CI=-5.68 to -0.50; P=0.02; Figure 6).

**Figure. 7:**
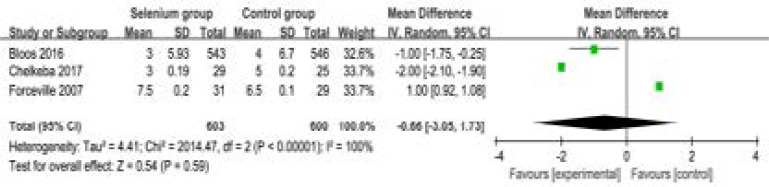
Forest plot for the meta-analysis of duration of vasopressor therapy (day).

**Figure. 8:**
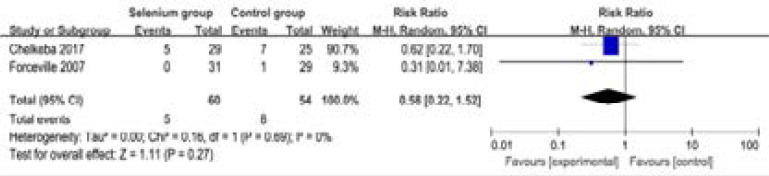
Forest plot for the meta-analysis of the incidence of acute renal failure.

**Figure. 9:**
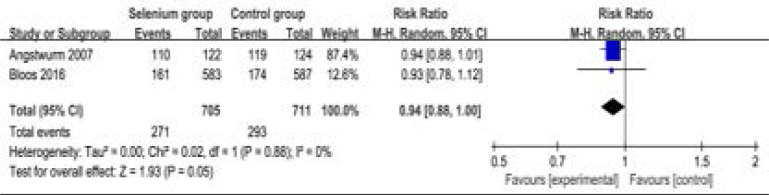
Forest plot for the meta-analysis of adverse events.

**Figure. 10:**
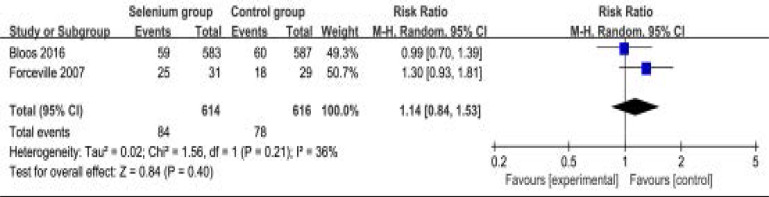
Forest plot for the meta-analysis of serious adverse events.

In addition, selenium administration results in no substantial influence on duration of vasopressor therapy (MD=-0.66; 95% CI=-3.05 to 1.73; P=0.59; Figure 7), the incidence of acute renal failure (RR=0.58; 95% CI=0.22 to 1.52; P=0.27; Figure 8), adverse events (RR=0.94; 95% CI=0.88 to 1.00; P=0.05; Figure 9) and serious adverse events (RR=1.14; 95% CI=0.84 to 1.53; P=0.40; Figure 10).

## Discussion

Our meta-analysis suggests that selenium administration is associated with significantly reduced all-cause mortality and length of hospital stay, but has no remarkable influence on 28-day mortality, length of intensive care unit (ICU) stay, duration of vasopressor therapy, and the incidence of acute renal failure in septic patients.

An overwhelming inflammation occurs in the initial phase of sepsis, during which migration of the neutrophils into the inflamed tissue releases free radicals that result in capillary congestion due to the damage of the endothelium and epithelium tissue, and respiratory failure due to leukocyte and macrophage infiltration into the site of inflammation of the respiratory system^14^, ^30^. Therapies counteracting the inflammation and oxidative stress remain attractive and challenging. Selenium is located on the catalytic center of most selenoenzymes, serves as an essential trace element for the biosynthesis and function of about 25 known selenocysteine containing selenoproteins 31. One of the best-known redox systems is glutathione complex consisting of the selenium-dependent peroxidases and the thioredoxin reductases 32, 33. Selenium has been reported to have an important role as anti-inflammatory agent by tightly regulating the expression of proinflammatory genes in immune cells 34, 35.

Some multiple-center trials confirm the efficacy of high-dose sodium selenite supplementation in patients with severe sepsis and septic shock to reduce of 28-day mortality 16, 36, 37. However, other clinical studies are not consistent with this results. The 28-day mortality is not decreased after selenium administration in septic patients and in critically ill patients 15, 20. One clinical study demonstrates that administration of selenium leads to a slight decrease in the duration of mechanical ventilation and length of ICU stay 38. In this meta-analysis, the mortality and length of hospital stay was confirmed to be reduced after selenium intervention in septic patients.

Patients are resuscitated with selenium initially, and end up with fewer recurrent pneumonia and sepsis, but they are weaned quicker due to cardioprotective properties of selenium against reperfusion and reoxygenation. Thus, selenium may increase sepsis-related cardiomyopathy and other complications 38. However, there is no increase in adverse events, and serious adverse events after selenium administration for septic patients based on the results of our meta-analysis.

The signaling pathways in inflammation and oxidative stress in vivo are quite complex during the sepsis, and there is always a cross talk between inflammation, oxidative stress and coagulation 39, 40. Regarding the effect of selenium on oxidative stress in septic patient, selenium administration is associated with a significant increase in the plasma activity glutathione peroxidase from day 3 to 14, but has no effect on the average plasma levels of inflammatory cytokines IL-6, IL-8 and IL-10 14, 41. The production and release of sepsis mediators is a complex network rather than as a cascade. Although one of the substances responsible for the initial phase is blocked, other mediators will likely maintain the septic response 42, 43. In 165 patients requiring mechanical ventilation for sepsis or septic shock, a study formula consists of high level of omega-3 fatty acids and γ-linolenic oil, less omega-6 fatty acids, and high doses of the antioxidant vitamins E and C and selenium, and patients receiving this study formula result in significant decrease in mortality and organ dysfunction, as well as the improvement in oxygenation, ventilator-free days and ICU-free days 44.

This meta-analysis has several potential limitations that should be taken into account. Firstly, our analysis is based on only five RCTs and three of them have a relatively small sample size (n<100). Overestimation of the treatment effect is more likely in smaller trials compared with larger samples. Next, the methods and duration of selenium administration are different in included RCTs, and may have an influence on the pooling results. Finally, some unpublished and missing data may lead bias to the pooled effect.

## Conclusion

Selenium administration may have the important ability to reduce mortality and length of hospital stay in patients with sepsis or septic shock, and selenium should be recommended to be administrated in clinical work with caution.
